# Growth Velocity and the Pubertal Growth Spurt Across Skeletal Maturity Stages in Class II Malocclusion: A Longitudinal Analysis

**DOI:** 10.3390/children12121612

**Published:** 2025-11-26

**Authors:** Nan-Hee Kim, Ji-Hyun Lee, Sanghee Lee, Yoon Jeong Choi, Chooryung Judi Chung, Kee-Joon Lee, Kyung-Ho Kim

**Affiliations:** 1 Department of Orthodontics, Institute of Craniofacial Deformity, Yonsei University College of Dentistry, Seoul 03722, Republic of Korea; 2 Department of Orthodontics, Institute of Craniofacial Deformity, Gangnam Severance Dental Hospital, Yonsei University College of Dentistry, Seoul 06273, Republic of Korea; 3 Department of Orthodontics, University of Washington, Seattle, WA 98105, USA

**Keywords:** Class II malocclusion, skeletal maturity, height growth velocity, peak height velocity, pubertal growth spurt, hand-wrist radiograph

## Abstract

**Background/Objectives**: This retrospective longitudinal study analyzed height growth velocity (GV) across skeletal maturity indicator (SMI) intervals in Korean adolescents with skeletal Class II malocclusion to identify the timing and duration of peak height velocity (PHV) and to define a PHV window relevant to growth-modification treatment. **Methods**: Sixty patients (30 males, 30 females) were followed for at least six years with serial hand-wrist radiographs and height records. GV was calculated for each SMI interval, defined as the period between adjacent SMI stages. In females, the timing of menarche by SMI stage was also recorded. **Results**: GV increased with skeletal maturation and was greatest at the SMI 5–6 interval in both sexes. In males, GV was already high at SMI 4–5 and did not differ significantly from that at SMI 5–6, whereas in females GV remained high at SMI 6–7 and did not differ significantly from that at SMI 5–6. In all female subjects, menarche occurred after PHV and coincided with growth deceleration. GV declined markedly after SMI 7–8, with minimal growth beyond SMI 11. **Conclusions**: These findings indicate that the PHV window spans SMI 4–6 in males and SMI 5–7 in females, suggesting that growth-modification therapy may achieve optimal outcomes when initiated within these stages. Because this study evaluated height rather than maxillomandibular growth and individual variation persists, SMI stage alone cannot define PHV, and additional assessments of growth are required when determining the timing of orthopedic intervention.

## 1. Introduction

Skeletal Class II malocclusion is a prevalent and clinically relevant dentofacial discrepancy in growing patients, typically resulting from mandibular deficiency, maxillary excess, or a combination of both. When mandibular deficiency is the primary contributing factor, orthopedic treatment with functional appliances during the growth period is widely regarded as an effective strategy for correcting skeletal imbalances [1,2,3]. This approach relies on the patient’s intrinsic growth potential; therefore, the timing of intervention plays a critical role in determining treatment success. Similar to the acceleration, peak, and deceleration phases observed in height growth during puberty, mandibular growth also follows a pubertal spurt pattern. Initiating treatment too early, before the acceleration phase, or too late, after the deceleration of growth, often results in suboptimal skeletal correction, as demonstrated in multiple clinical and systematic studies [4,5,6]. The pubertal growth spurt—when mandibular growth reaches its peak—has been identified as the most favorable period for orthopedic intervention [7,8]. Therefore, in the growth modification of skeletal Class II malocclusion associated with mandibular deficiency, accurately identifying the peak mandibular growth period is of paramount importance to maximize treatment efficacy [4,7,8].

Identifying the peak growth periods of the jaws in clinical settings remains inherently challenging, as direct evaluation of mandibular growth requires serial imaging and entails concerns about radiation exposure. Moreover, by the time the true peak of mandibular growth is recognized, the optimal window for orthopedic intervention may already have passed. Consequently, researchers have sought surrogate indicators that reflect jaw development patterns. Among these, height has been widely employed because it represents overall skeletal growth, is easy to measure, and is readily understood by both clinicians and caregivers [9,10,11]. Peak height velocity (PHV) has been reported to occur either slightly before or concurrently with the peak of craniofacial growth [12,13], suggesting a close temporal relationship between overall skeletal and jaw growth. In particular, longitudinal investigations have demonstrated that the timing of PHV closely coincides with the period of maximal mandibular growth during adolescence [9,11,14], supporting its value as a clinically useful reference for determining the optimal timing of orthopedic intervention. Although no single biological marker can precisely identify the onset of the mandibular growth peak, PHV remains a practical and noninvasive proxy owing to its temporal correspondence with craniofacial skeletal development [13,14,15].

Chronological age is insufficient to capture individual variability in growth potential, as it fails to account for differences arising from sex, ethnicity, or generation. Instead, skeletal maturity has become a more biologically valid indicator of developmental status. Among various assessment modalities, hand-wrist radiography is particularly valued for its diagnostic reliability and reproducibility [14,15,16,17,18,19,20]. The Skeletal Maturity Indicators (SMI) system proposed by Fishman [15], which divides ossification stages of specific hand-wrist bones into eleven sequential phases, has become one of the most practical and widely adopted methods in orthodontic diagnosis and treatment planning.

Previous investigations examining the relationship between skeletal maturity indicator (SMI) stages and height growth velocity have demonstrated a generally consistent pattern across decades. Although some variation exists among studies, most have reported peak height velocity (PHV) between SMI stages 5 and 7.

Fishman [15] suggested that PHV occurs around SMI 6 in males and SMI 5 in females, while Hägg and Taranger [14] reported PHV around SMI 6 in both sexes. Later research in Korean adolescents by Kim and Lee [21] identified PHV most frequently at SMI 6–7 in females and SMI 5–7 in males, suggesting that the timing of PHV may vary across populations, possibly reflecting ethnic or generational differences in pubertal development.

Hwang et al. [22] compared Class I and Class III patients and observed PHV at SMI 5–6 in males and SMI 6–7 in females, with Class III subjects showing a delayed and prolonged growth peak. Ghaleb et al. [23] reported that the pubertal growth spurt lasted longer in Class II than in Class I subjects, whereas Stahl et al. [24] found reduced mandibular growth during the peak period in untreated Class II patients. Collectively, these findings indicate that the growth spurt pattern in skeletal Class II malocclusion may differ from that of other malocclusion types. However, few studies have directly examined this relationship.

The present study addresses this gap by conducting a retrospective longitudinal analysis of height-growth velocity across SMI stages in Korean adolescents with skeletal Class II malocclusion. Using existing hand-wrist radiographs and serial height records obtained from patients undergoing growth monitoring, this study aims to identify the SMI stages corresponding to PHV and to provide clinically relevant reference points for determining the optimal timing of growth modification therapy in patients with mandibular deficiency.

## 2. Materials and Methods

### 2.1. Study Design and Participants

This retrospective longitudinal study reviewed 1086 patients (532 males and 554 females) diagnosed with malocclusion who had longitudinal growth-monitoring records and no history of congenital craniofacial anomalies or growth-hormone therapy. Only radiographic data obtained after 2006 were analyzed to ensure the use of standardized digital hand-wrist radiographs with consistent image quality suitable for longitudinal evaluation.

The study protocol was approved by the Institutional Review Board of Yonsei University Gangnam Severance Hospital (IRB No. 3-2024-0135), and the requirement for informed consent was waived due to the retrospective design.

Skeletal Class II malocclusion was defined as an ANB angle ≥ 5°, a conservative threshold chosen to ensure clear and reproducible identification of skeletal discrepancies. Among the reviewed patients, 131 males and 121 females met this criterion.

Although the ANB angle may be influenced by cranial-base length, nasion position, or mandibular rotation, it was adopted as the primary diagnostic criterion because it provides a clinically established and reproducible sagittal reference for baseline classification. No restrictions were placed on vertical pattern or dental compensation in order to include a clinically representative range of skeletal Class II patients.

Among the skeletal Class II patients, 97 males and 64 females had at least 12 serial hand-wrist radiographs with corresponding height measurements collected over a minimum 6-year observation period.

A total of 42 males and 51 females had reached final height, with no further increase observed in serial records. Menarche data were available for 38 female subjects based on self or parental report.

Because this study aimed to analyze height-growth changes across all SMI stages (1–11), only subjects in whom every SMI stage could be clearly identified were included in the final sample. This inclusion criterion may have introduced a degree of selection bias toward individuals with more complete longitudinal records; however, this was addressed by focusing on within-subject changes across SMI intervals rather than on absolute cross-sectional comparisons.

**Figure 1:**
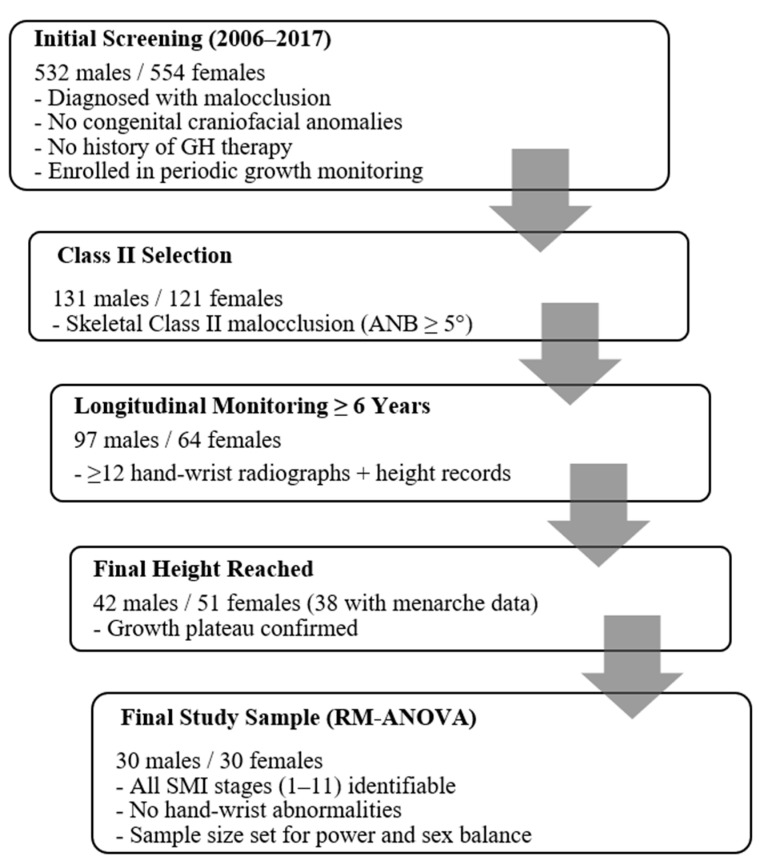
Flowchart of participant selection for the longitudinal sample. The diagram summarizes the stepwise inclusion process used to identify the final cohort (30 males and 30 females) with complete SMI stage records and longitudinal height measurements spanning SMI stages 1–11. Abbreviation: RM-ANOVA, repeated-measures analysis of variance.

The final analysis included 60 subjects (30 males and 30 females) with normal hand-wrist morphology. The participant-selection process is summarized in Figure 1. The average measurement interval was 7.1 months for males and 6.9 months for females.

### 2.2. Measurements

Skeletal maturity was evaluated from standardized digital hand-wrist radiographs using Fishman’s SMIs [15]. All radiographs were obtained under identical exposure settings and positioning to ensure consistency. Ossification stages observed prior to SMI 1 were designated as SMI 0. Each radiograph was analyzed twice by the same examiner at two-week intervals to confirm intra-observer reliability. Only radiographs with clearly identifiable anatomical landmarks were included, and no significant differences were found between repeated measurements.

Height was measured at every hand-wrist radiographic examination on the same day, using a calibrated stadiometer. Chronological age was recorded at each measurement, allowing direct matching of height data with the corresponding SMI stage. For each participant, the height and age corresponding to the first identification of each SMI stage were used for analysis.

**Figure 2:**
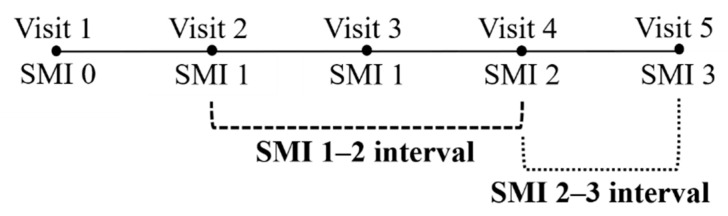
Example of how SMI intervals were defined. This schematic illustrates the method used to determine SMI intervals from consecutive visits. In this example, Visit 1 shows SMI 0, followed by repeated SMI 1 values at Visits 2 and 3, and subsequent progression to SMI 2 and 3. When three consecutive measurements showed SMI values of 1, 1, and 2, the corresponding interval was defined as SMI 1–2. The first appearance of each SMI stage was used to assign the interval, and this approach was consistently applied throughout the study.

For longitudinal evaluation, the interval between consecutive SMI stages was defined as the SMI interval, representing the time span between the first observation of a given SMI stage and the subsequent stage (e.g., SMI 1–2 refers to the period from the first appearance of SMI 1 to that of SMI 2; Figure 2). This operational definition allowed consistent segmentation of skeletal maturation periods for longitudinal growth monitoring.

Growth patterns across SMI intervals were assessed using three indices: growth velocity (GV), relative growth rate (RGR), and percentage of growth attained (% Growth Attained).

GV was defined as the increase in height during each SMI interval divided by the time in years, allowing identification of the interval corresponding to the peak height velocity (PHV).

RGR represented the proportion of height gained during each interval relative to the total height increase between SMI 1 and 11, calculated as

$${\mathrm{RGR}}_{n\to n+1}=\frac{H_{n+1}-H_{n}}{H_{\left(\mathrm{first}\;\mathrm{SMI}\;11\right)}-H_{\left(\mathrm{SMI}\;1\right)}}\times 100$$

where $H_{n}$ and $H_{n+1}$ represent the heights at the first identification of SMI stage n and n+1, respectively.

The percentage of growth attained (% Growth Attained) was calculated to indicate cumulative growth progress from SMI 1 up to a given stage, as a proportion of total growth between SMI 1 and 11:

$$\%Growth\;Attained=\frac{H_{\mathrm{current}}-H_{\mathrm{SMI}\;1}}{H_{\mathrm{first}\;\mathrm{SMI}\;11}-H_{\mathrm{SMI}\;1}}\times 100$$

This parameter reflects an individual’s cumulative growth pattern and corresponds to the cumulative sum of RGRs across SMI intervals.

In female subjects, the relationship between menarche and skeletal maturity (SMI) was also examined. Menarche timing was obtained by self or parental report, and the corresponding SMI stage was estimated from the hand-wrist radiograph taken closest to the reported menarche date. The interval between menarche and PHV, as well as post-menarcheal growth, was also evaluated.

### 2.3. Statistical Analysis

All statistical analyses were performed separately for males and females. Changes in growth velocity (GV) across skeletal maturity indicator (SMI) intervals were first examined using repeated-measures analysis of variance (RM-ANOVA) to determine whether GV differed significantly across stages. When RM-ANOVA indicated a significant overall effect, post hoc pairwise comparisons between adjacent SMI intervals were performed using paired *t*-tests. Because the primary clinical objective of this study was to detect meaningful stage-to-stage transitions rather than to control for family-wise Type I error across all possible comparisons, unadjusted *p*-values were reported for these adjacent-interval analyses.

To further account for within-subject correlations in the longitudinal dataset, a linear mixed-effects model was additionally constructed with GV as the dependent variable, SMI interval as a fixed effect, and subject ID as a random intercept. Estimated marginal means (EMMs) for each SMI interval were derived from the fitted model using the emmeans framework, and Tukey-adjusted pairwise contrasts between EMMs were computed to aid interpretation of longitudinal trends and to confirm the interval corresponding to peak height velocity (PHV).

Sex differences in GV, chronological age, and percentage of growth attained at each SMI interval were examined using independent-samples *t*-tests.

All analyses were performed using R software (version 4.3.1; R Foundation for Statistical Computing, Vienna, Austria). Linear mixed-effects models were fitted using the *lme4* package, and EMMs with post hoc contrasts were generated using the emmeans package. Statistical significance was defined as *p* < 0.05 for all analyses.

## 3. Results

### 3.1. GV and Timing of PHV

At baseline, the mean ANB was 6.4° ± 1.3 (range, 5.2–9.6°) in males and 8.0° ± 1.0 (range, 6.0–10.0°) in females. Adult height averaged 174.7 ± 6.6 cm (range, 161.2–190.4 cm) in males and 160.8 ± 5.3 cm (range, 147.2–170.1 cm) in females. The chronological ages at which each SMI stage was first observed are summarized in Table 1. Females were significantly younger than males at all stages (*p* < 0.01).

**Figure 3:**
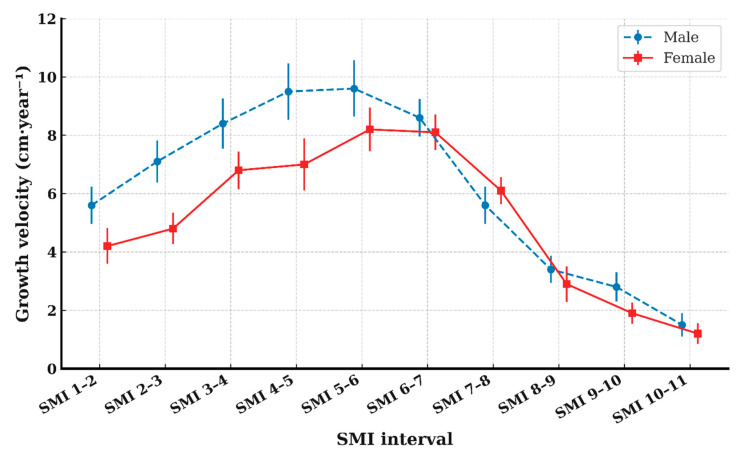
Mean height growth velocity across SMI intervals, showing a common peak at the SMI 5–6 interval. Lines indicate the mean growth velocity (GV) for males and females; error bars represent 95% confidence intervals. Data points for each sex were horizontally offset to reduce overlap.

GV by SMI interval are presented in Table 2 and Table 3. Repeated-measures ANOVA demonstrated significant differences in GV across SMI intervals in both sexes (*p* < 0.001). GV showed a gradual increase from SMI 1–2 through SMI 5–6, followed by a progressive decline, as illustrated in Figure 3. GV remained relatively high at SMI 6–7 but decreased markedly thereafter, particularly after SMI 7–8.

In males, GV at SMI 4–5 and 5–6 was comparable, and in females, GV at SMI 5–6 and 6–7 showed a similarly high level; pairwise comparisons (Table 4) confirmed that these adjacent intervals did not differ significantly. Based on these findings, the high-growth period was estimated to span SMI 4–6 in males and SMI 5–7 in females, corresponding to chronological ages of approximately 12.0–13.0 years and 10.9–11.8 years, respectively.

RGR analysis further confirmed sex differences (Table 5). In males, RGR increased from 9.4% at SMI 1–2 to 13.8% at SMI 3–4 and remained stable through SMI 4–7, but declined sharply after SMI 8–9. In females, RGR rose to 12.7% at SMI 3–4 and peaked at 15.9% at SMI 7–8, followed by a steep decline.

### 3.2. Individual Variation in the Timing of PHV

**Figure 4:**
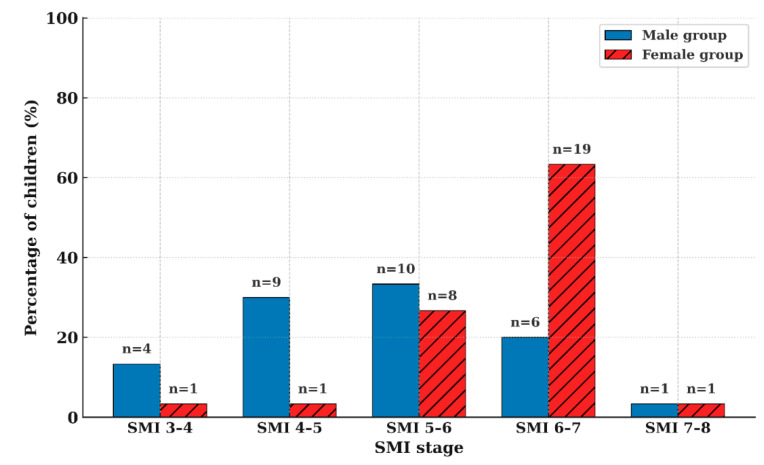
Distribution of individual PHV timing across SMI intervals in males and females, demonstrating marked inter-individual variation.

Although the mean GV peaked at the SMI 5–6 interval at the group level, individual PHV—defined as each subject’s peak annual height velocity—did not consistently occur at this stage. Instead, the timing of PHV was distributed across a wide range of SMI stages, demonstrating substantial inter-individual variability (Figure 4).

Individual PHV occurred across a wide range of SMI stages, from SMI 3–4 to 7–8 (Figure 4). Among males, 63.3% exhibited PHV between SMI 4–6, whereas 90.0% of females exhibited PHV between SMI 5–7 (*p* < 0.01). The individual peak annual height increment was 11.5 ± 1.9 cm·year^−1^ in males (range, 8.0–16.3 cm·year^−1^) and 9.2 ± 1.3 cm·year^−1^ in females (range, 7.2–12.0 cm·year^−1^) (*p* < 0.001). Height attained at PHV corresponded to 91.5 ± 3.0% of adult height in males and 92.1 ± 2.3% in females, showing no significant sex-related difference (*p* > 0.05).

Despite this substantial inter-individual variation, group-level analyses revealed a highly consistent developmental pattern when growth velocity was examined across SMI intervals.

The linear mixed-effects model demonstrated that SMI interval was a significant fixed effect on GV in both sexes (males: *p* < 0.001; females: *p* < 0.001). Estimated marginal means (EMMs) showed that the highest predicted GV occurred at the SMI 5–6 interval in both males and females, confirming this stage as corresponding to peak height velocity (PHV).

In males, the EMM at SMI 5–6 was the highest, but it was not significantly different from that at SMI 4–5 (Tukey–adjusted *p* > 0.05), indicating a broad peak spanning SMI 4–6. In females, the EMM at SMI 5–6 also represented the peak value, and no significant difference was found between SMI 5–6 and 6–7 (Tukey–adjusted *p* > 0.05), suggesting an extended peak from SMI 5–7.

Beyond the PHV interval, predicted GV declined sharply, beginning at SMI 6–7 in males and SMI 7–8 in females, with significantly lower GV from SMI 8–9 onward in both sexes (all Tukey–adjusted *p* < 0.01).

### 3.3. Growth Deceleration and Growth Completion

**Figure 5:**
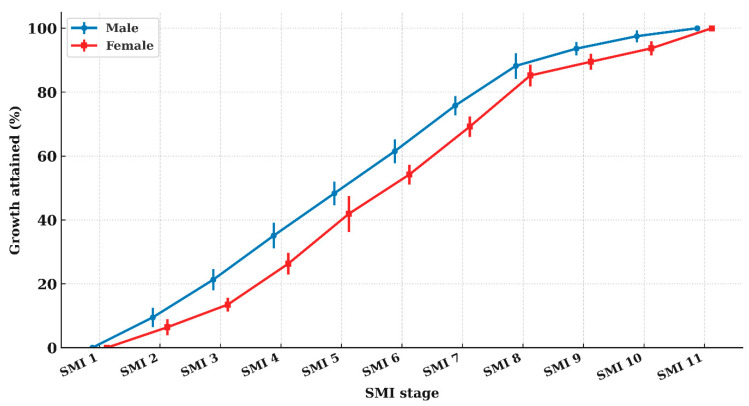
Cumulative percentage of growth attained (% Growth Attained) by SMI stage. For normalization, SMI 1 was set to 0% and SMI 11 to 100%, and the cumulative growth at each stage was evaluated. Lines show mean values with standard deviations (SD). Both sexes show a reduced slope after the SMI 7–8 stage, indicating deceleration toward the end of pubertal height growth.

Figure 5 illustrates cumulative height gain across SMI intervals, assuming a 100% total growth from SMI stage 1 to 11 (i.e., from the first observation of SMI 1 to the first observation of SMI 11). As skeletal maturity advanced, cumulative height increased steadily in both sexes. At SMI 7–8, growth velocity declined, with 88.1% and 85.1% of adult height attained in males and females, respectively, indicating limited remaining growth.

**Figure 6:**
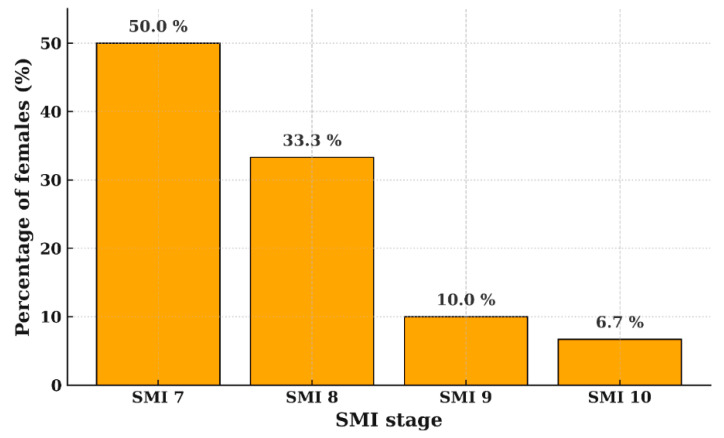
SMI stage observed closest to menarche in females, showing that most subjects were in SMI 7 or 8.

Menarche occurred at a mean age of 12.5 ± 1.0 years (range, 10.8–14.2 years). The time interval between PHV and menarche ranged 3–34 months (mean 1.1 ± 0.8 years), and no participants experienced menarche before PHV. Figure 6 shows the SMI stage closest to menarche, with 83.3% of females reaching menarche at SMI 7–8 and none before stage 7. The residual growth rate was estimated as 3.4 ± 1.3% of adult height (range, 0.7–6.7%), corresponding to a mean additional height gain of 5.5 ± 2.6 cm (range, 1.0–10.4 cm).

A few individuals grew after SMI 11; however, the additional gain was minimal. The average age at SMI stage 11 was 16.1 ± 0.8 years (range, 15.2–18.0) in males and 14.8 ± 1.1 years (range, 12.9–17.3) in females. Following this stage, the mean height gain was 1.2 ± 1.0 cm (range, 0–4.0 cm) in males and 0.7 ± 0.6 cm (range, 0–1.8 cm) in females. The amount of growth after SMI stage 11 and the final adult height differed significantly between sexes (*p* < 0.05).

## 4. Discussion

This longitudinal study examined the timing of peak height velocity (PHV) in Korean adolescents with skeletal Class II malocclusion using serial height measurements and hand-wrist skeletal maturity indicators (SMI). The greatest mean growth velocity occurred at the SMI 5–6 interval in both sexes. However, males exhibited similarly high velocity at SMI 4–5, whereas females showed a comparable level at SMI 6–7. Pairwise comparisons between adjacent SMI intervals (Table 4) confirmed that these neighboring intervals were not significantly different within each sex, indicating that the clinically meaningful high-growth period spans SMI 4–6 in males and SMI 5–7 in females. This sex-specific distribution of growth intensity provides a more nuanced interpretation than relying solely on the single highest GV value.

When interpreted within the broader context of craniofacial development, these findings reinforce the rationale for using skeletal maturity to guide treatment timing in skeletal Class II malocclusion. Mandibular growth accelerates sharply around the pubertal peak and contributes substantially to the reduction in facial convexity during adolescence [13,25,26]. Functional appliances that aim to enhance mandibular projection, such as the Twin Block, show their greatest skeletal effects when used during this rapid-growth window [27,28]. The present results suggest that this window aligns approximately with SMI 4–6 in males and SMI 5–7 in females. In contrast, interventions targeting maxillary excess are optimally timed earlier [25,29,30]. For adolescents who present with both maxillary excess and mandibular deficiency, a staged approach—early maxillary control followed by peak-phase mandibular enhancement—may achieve more balanced skeletal correction.

Because both the present study and the work of Hwang et al. [22] examined Korean adolescents, comparison across malocclusion types provides insight into potential population-specific differences in pubertal growth patterns. Hwang et al. [22] reported that PHV occurred at SMI stages 5–6 in males and 6–7 in females, with Class III adolescents showing a tendency toward a delayed and more prolonged pubertal peak compared with Class I subjects. In the present Class II cohort, the mean GV peaked at SMI 5–6 in both sexes; however, the surrounding high-growth window differed slightly between sexes (SMI 4–6 in males and 5–7 in females). Taken together, these findings suggest that the timing and duration of the pubertal growth spurt may vary across skeletal classifications—Class I, II, and III—rather than following a uniform maturational pattern within a single population. Because this study assessed height growth rather than maxillomandibular skeletal maturation, direct conclusions regarding jaw-specific growth timing cannot be drawn. Further longitudinal studies incorporating direct craniofacial measurements are warranted to determine whether true skeletal differences exist among malocclusion types and whether such differences should guide tailored orthopedic treatment timing.

Marked sex-related differences in growth velocity patterns were also evident. Males exhibited a more gradual acceleration-deceleration curve, whereas females showed a more concentrated peak occurring at an earlier age. These observations align with the well-established sexual dimorphism in pubertal maturation, wherein females undergo a shorter and more abrupt growth spurt while males follow a more prolonged trajectory [31,32,33]. Clinically, these distinctions underscore the need for sex-specific interpretation when determining the timing of growth modification: males may benefit from a broader therapeutic window spanning several SMI stages, whereas females require more precise timing due to their narrower and more rapidly progressing peak interval.

Substantial interindividual variability was observed in the timing of PHV. Although mean GV peaked at the SMI 5–6 interval at the population level, individual PHV did not consistently occur at this stage. Instead, individual PHV timing ranged widely—from SMI 3–4 to SMI 7–8—indicating that no single SMI interval can serve as a definitive marker of the peak for all individuals. This biological variability highlights a known limitation of skeletal maturity indicators: although SMI provides a practical developmental reference, it cannot fully capture the heterogeneity of pubertal growth patterns [34]. Accordingly, PHV assessment should incorporate multiple dimensions of growth, including skeletal maturity, chronological age, dental development, and longitudinal height records, rather than relying on a single indicator.

Recent advances in artificial intelligence offer additional promise in addressing these limitations. Deep-learning models have demonstrated high accuracy in automated assessment of SMI stages [35] and bone age estimation [36], and AI-based diagnostic frameworks are increasingly being incorporated into orthodontic growth prediction and treatment planning [37]. As longitudinal datasets linking SMI, craniofacial morphology, and treatment outcomes continue to expand, AI-driven analytic tools may complement conventional biologic maturity indicators and support more individualized prediction of jaw-specific growth potential in skeletal Class II patients.

Following the SMI 7–8 interval, both growth velocity and remaining height gain declined sharply. Approximately 85% of total height gain from SMI stages 1–11 had already been achieved by SMI stage 8. In females, menarche occurred exclusively after the peak growth period, with no subjects reporting menarche before SMI stage 7. This reaffirms its role as a post-peak developmental indicator [14,38,39]. These findings collectively indicate that once patients progress beyond SMI 7–8, the biological potential for meaningful skeletal modification becomes markedly limited. Growth modification therapy should therefore be initiated no later than SMI stage 7 to maximize orthopedic responsiveness.

This study should be interpreted in light of several limitations. As a retrospective analysis, hand-wrist radiographs and height measurements were obtained at average intervals of 7.1 months in males and 6.9 months in females, with occasional deviations of 1–2 months due to variable follow-up schedules. External factors such as nutrition, lifestyle, and physical activity were not controlled and may have influenced individual growth trajectories.

Additionally, this single-center sample included only skeletal Class II patients, limiting generalizability to other skeletal patterns or populations. Despite these limitations, the present study provides a robust longitudinal assessment of height growth velocity across SMI stages and identifies the maturity intervals most closely associated with PHV. These findings offer clinically meaningful guidance regarding the timing of growth modification in skeletal Class II malocclusion and highlight the importance of integrating skeletal maturity staging with individualized growth monitoring when planning orthopedic interventions.

## 5. Conclusions

This longitudinal analysis demonstrated that the greatest mean height velocity occurred at the SMI 5–6 interval in both sexes. However, the surrounding intervals showed sex-specific patterns: males exhibited similarly high growth at SMI 4–5, whereas females maintained elevated growth at SMI 6–7. These findings indicate that the clinically meaningful PHV window is centered around SMI stage 5 in males and stage 6 in females. Accordingly, the period of greatest growth potential appears to span SMI 4–6 in males and SMI 5–7 in females, suggesting that growth modification therapy—particularly mandibular enhancement—may achieve optimal outcomes when initiated within these stages.

It must be noted, however, that this study assessed height growth rather than maxillomandibular growth directly. Although SMI provides a practical indicator of maturation, considerable inter-individual variation persists, and PHV cannot be precisely inferred from skeletal stage alone. A comprehensive, multidimensional evaluation of growth is therefore essential when determining the optimal timing for orthopedic intervention.

## Figures and Tables

**Table 1:** Chronological age distribution by SMI stage in males and females.

SMI Stage	Male	Female	*p*
1	10.2 ± 0.7	9.0 ± 0.7	**
2	10.8 ± 0.8	9.4 ± 0.8	**
3	11.4 ± 0.9	9.8 ± 0.8	**
4	12.0 ± 0.8	10.3 ± 0.7	**
5	12.5 ± 0.8	10.9 ± 0.8	**
6	13.0 ± 0.8	11.3 ± 0.9	**
7	13.6 ± 0.8	11.8 ± 0.9	**
8	14.4 ± 0.7	12.5 ± 0.9	**
9	15.0 ± 0.7	12.9 ± 0.8	**
10	15.5 ± 0.7	13.5 ± 0.8	**
11	16.1 ± 0.8	14.8 ± 1.1	**

SMI, skeletal maturity indicator; ** *p* < 0.01.

**Table 2:** Growth velocity across SMI intervals in male subjects.

SMI Interval	GV (cm·Year^−1^)		
	Mean	SD	95% CI
1–2	5.6	1.8	5.0–6.2
2–3	7.1	2.0	6.4–7.8
3–4	8.4	2.4	7.5–9.3
4–5	9.5	2.7	8.5–10.5
5–6	9.6	2.7	8.6–10.6
6–7	8.6	1.8	8.0–9.2
7–8	5.6	1.8	5.0–6.2
8–9	3.4	1.3	2.9–3.9
9–10	2.8	1.4	2.3–3.3
10–11	1.5	1.1	1.1–1.9

SMI, skeletal maturity indicator; GV, growth velocity; SD, standard deviation; 95% CI, 95% confidence interval.

**Table 3:** Growth velocity across SMI intervals in female subjects.

SMI Interval	GV (cm·Year^−1^)		
	Mean	SD	95% CI
1–2	4.2	1.7	3.6–4.8
2–3	4.8	1.5	4.3–5.3
3–4	6.8	1.8	6.2–7.4
4–5	7.0	2.5	6.1–7.9
5–6	8.2	2.1	7.4–9.0
6–7	8.1	1.7	7.5–8.7
7–8	6.1	1.3	5.6–6.6
8–9	2.9	1.7	2.3–3.5
9–10	1.9	1.0	1.5–2.3
10–11	1.2	1.0	0.8–1.6

SMI, skeletal maturity indicator; GV, growth velocity; SD, standard deviation; 95% CI, 95% confidence interval.

**Table 4:** Pairwise comparisons of growth velocity between adjacent SMI intervals.

Comparison (SMI Interval)	Male		Female	
	*t*	*p* (raw)	*t*	*p* (raw)
SMI 1–2 → 2–3	−3.46	0.0017	−0.92	0.366
SMI 2–3 → 3–4	−3.40	0.0020	−6.67	2.56 × 10^−7^
SMI 3–4 → 4–5	−3.34	0.0023	−0.71	0.481
SMI 4–5 → 5–6	−0.22	0.825	−3.92	4.98 × 10^−4^
SMI 5–6 → 6–7	2.17	0.038	0.82	0.417
SMI 6–7 → 7–8	7.53	2.70 × 10^−8^	4.70	5.79 × 10^−5^
SMI 7–8 → 8–9	7.79	1.35 × 10^−8^	8.08	6.57 × 10^−9^
SMI 8–9 → 9–10	3.02	0.0052	3.26	0.0029
SMI 9–10 → 10–11	4.27	0.00019	3.65	0.00103

Notes. Paired *t*-tests were used to compare growth velocity (GV) between adjacent skeletal maturity indicator (SMI) intervals within each sex. Unadjusted *p*-values are reported because the clinical objective was to identify meaningful stage-to-stage transitions rather than to control the family-wise Type I error rate. Negative *t*-values indicate an increase in GV from the earlier to the later interval, whereas positive values indicate a decrease. Statistical significance was defined as *p* < 0.05.

**Table 5:** Relative growth rate across SMI intervals.

SMI Interval	Male			Female		
	Mean	SD	95% CI	Mean	SD	95% CI
1–2	9.4	3.0	8.3–10.5	6.3	2.5	5.4–7.2
2–3	11.8	3.3	10.6–13.0	7.2	2.2	6.4–8.0
3–4	13.8	4.0	12.4–15.2	12.7	3.4	11.5–13.9
4–5	13.2	3.7	11.9–14.5	15.7	5.6	13.7–17.7
5–6	13.2	3.7	11.9–14.5	12.2	3.1	11.1–13.3
6–7	14.3	3.0	13.2–15.4	15.1	3.2	14.0–16.2
7–8	12.4	4.0	11.0–13.8	15.9	3.4	14.7–17.1
8–9	5.5	2.1	4.7–6.3	4.3	2.5	3.4–5.2
9–10	3.9	1.9	3.2–4.6	4.3	2.2	3.5–5.1
10–11	2.5	1.8	1.9–3.1	5.8	4.9	4.0–7.6

SMI, skeletal maturity indicator; SD, standard deviation; 95% CI, 95% confidence interval.

## Data Availability

The data are not publicly available due to patient privacy and ethical restrictions, but may be obtained from the corresponding author upon reasonable request.
